# New Insights into Immune-Mediated Mechanisms in Parkinson’s Disease

**DOI:** 10.3390/ijms21239302

**Published:** 2020-12-06

**Authors:** Jolene Su Yi Tan, Yin Xia Chao, Olaf Rötzschke, Eng-King Tan

**Affiliations:** 1 Duke-NUS Medical School, Singapore 169857, Singapore; e0011424@u.duke.nus.edu (J.S.Y.T.); chao.yinxia@singhealth.com.sg (Y.X.C.); 2 Department of Neurology, National Neuroscience Institute of Singapore, Singapore General Hospital Campus, Singapore 169856, Singapore; 3 Singapore Immunology Network (SIgN), A*STAR, Singapore 138648, Singapore

**Keywords:** Parkinson’s disease, neuroinflammation, innate and adaptive immunity, gut-brain axis

## Abstract

The immune system has been increasingly recognized as a major contributor in the pathogenesis of Parkinson’s disease (PD). The double-edged nature of the immune system poses a problem in harnessing immunomodulatory therapies to prevent and slow the progression of this debilitating disease. To tackle this conundrum, understanding the mechanisms underlying immune-mediated neuronal death will aid in the identification of neuroprotective strategies to preserve dopaminergic neurons. Specific innate and adaptive immune mediators may directly or indirectly induce dopaminergic neuronal death. Genetic factors, the gut-brain axis and the recent identification of PD-specific T cells may provide novel mechanistic insights on PD pathogenesis. Future studies to address the gaps in the identification of autoantibodies, variability in immunophenotyping studies and the contribution of gut dysbiosis to PD may eventually provide new therapeutic targets for PD.

## 1. Introduction

Parkinson’s disease (PD) is one of the most common neurodegenerative disorders, affecting more than 6 million people worldwide [1,2,3]. It is neuropathologically characterized by the loss of dopaminergic neurons in the substantia nigra (SN) and the presence of Lewy bodies containing aggregates of α-synuclein [4]. PD patients primarily present motor symptoms such as rigidity, tremors and bradykinesia and a broad range of non-motor symptoms during the prodromal phase. These non-motor symptoms, which include cognitive impairment, sleep disturbances, psychiatric disorders and autonomic dysfunction, significantly impact the patient’s quality of life over the long term [5,6,7]. More importantly, early identification of PD by these non-motor symptoms might be critical, as the onset of motor symptoms in PD coincides with an irreversible loss of approximately 30–70% dopaminergic neurons in the SN [8,9]. Despite decades of research on the complex interplay between genetic and environmental risk factors in PD, the exact etiopathogenesis of PD has yet to be elucidated. Notably, neuroinflammation and autoimmune mechanisms are increasingly recognized in PD pathogenesis. These dysregulated processes are controlled by our immune system, which has vital roles in protecting us from noxious stimuli. The failure to achieve a delicate balance between pro- and anti-inflammatory immune players and the maintenance of self-tolerance predisposes one to a proinflammatory milieu [10,11]. Epidemiological studies have revealed an increased risk of PD development in patients with autoimmune diseases and the clinical observation of postencephalitic parkinsonism secondary to viral infection support the role of the immune system in PD progression [12,13]. The proinflammatory milieu in PD patients is validated peripherally and centrally through biochemical, immunophenotyping and post-mortem studies. PD patients have elevated proinflammatory cytokines such as interferon (IFN) γ, interleukin (IL)-1β, IL-2, IL-6 and tumor necrosis factor α (TNFα) in the blood and cerebrospinal fluid (CSF), which contribute to neuronal death [14,15]. Immune cell dysregulation and perturbations in immune subsets in PD patients through immunophenotyping reveal intrinsic changes, leading to an inflammatory predisposition [16,17]. The central involvement of immune mediators arises from the breakdown of the blood brain barrier (BBB), which protects the brain from peripheral immune cells [18]. This results in the presence of complement proteins, human leukocyte antigen DR isotype (HLA-DR) positive microglia, inflammasome activation, infiltration of CD4+ and CD8+ T lymphocytes and autoantibodies against neuronal antigens in the SN of PD patients [19,20,21]. Despite the wealth of studies implicating neuroinflammation in PD, the precise molecular mechanism leading to dopaminergic neuronal death through immune pathways remains unclear. By focusing on immune-mediated pathways, this review aims to integrate and highlight novel mechanisms involving genes, the gut-brain axis and innate and adaptive immune responses that may be involved in neurodegeneration. Through this, we hope to inspire a targeted search of PD inflammatory biomarkers and immune cells that aids in the mitigation of disease progression and prognostication.

## 2. Mechanisms of Neuronal Death: Innate Immunity

**Figure 1:**
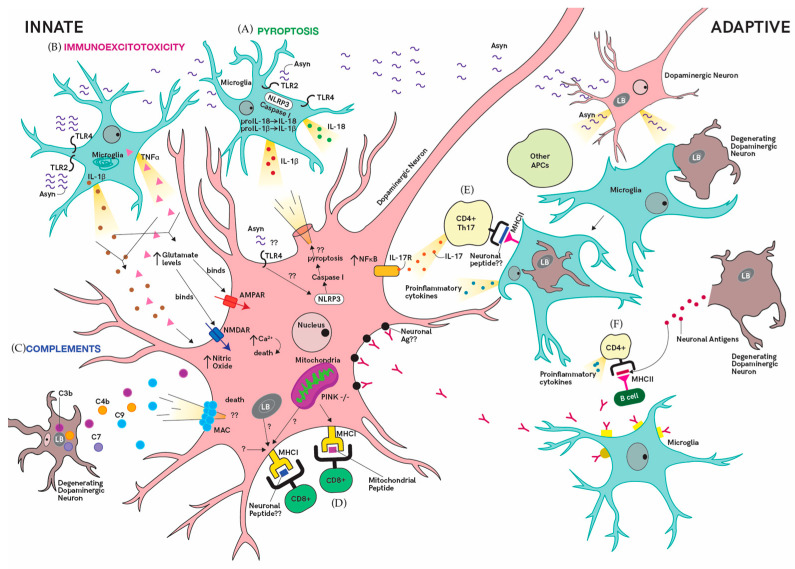
Mechanisms of immune-mediated neurotoxicity in dopaminergic neurons. Innate mechanisms of neurodegeneration are mediated by pyroptosis, immunoexcitotoxicity and the complement system. (**A**) Pyroptosis: α-synuclein (Asyn) activates microglia through its association with the toll like receptor 2 (TLR2) and 4 (TLR4), inducing inflammasome activation. The cytosolic nod like receptor protein 3 (NLRP3) inflammasome is formed and mediates caspase 1 activation. Caspase 1 cleaves pro-interleukin 1β (pro IL-1β) and pro-interleukin 18 (pro-IL-18) to IL-1β and IL-18, respectively, which will be released into the brain’s microenvironment. Dopaminergic neurons which express toll like receptor 4 (TL4) may allow α-synuclein to associate with it, leading to inflammasome activation in dopaminergic neurons. (**B**) Immunoexcitotoxicity: Microglia activation in the presence of α-synuclein induces the release of proinflammatory cytokines such as interleukin-1β (IL-1β) and tumor necrosis factor α (TNFα). These cytokines induce neuroexcitotoxicity by modulating the receptor density of excitatory receptors α-amino-3-hydroxy-5-methyl-4-isoxazolepropionic acid receptor (AMPAR) and N-methyl-D-aspartic acid or N-methyl-D-aspartate receptor (NMDAR) and glutamate levels. This causes excessive intracellular calcium flux, enhancing dopaminergic neurons susceptibility to neuronal death. Nitric oxide levels within the dopaminergic neuron are also increased in the presence of the proinflammatory cytokines. (**C**) Complement system: Complement components, C3b, C4b and C7 are present on Lewy bodies (LB) in dopaminergic neurons. Complement components may induce neuronal death through the formation of the membrane attack complex (MAC), leading to neuronal lysis. Adaptive mechanisms of neurodegeneration are mediated by CD4+ T cells, CD8+ T cells and B lymphocytes. (**D**) T cell-mediated neurotoxicity may arise directly from the interaction of cytotoxic CD8+ T cells with peptides presented on the major histocompatibility class I (MHC I). In the presence of phosphatase and tensin homolog (PTEN)-induced kinase 1 (*PINK1*) knockout with intestinal infection, there will be mitochondrial antigen presentation and the presence of mitochondrial-specific CD8+ T cells. Other PD-specific peptides presented on the MHC I of dopaminergic neurons that come from the LB, neuronal cell or mitochondria may be recognized by CD8+ T cells, and further investigation is required. (**E**) CD4+ T lymphocytes are activated in the presence of antigen presenting cells (APC) with peptides presented on the major histocompatibility class II (MHC II). These peptides are derived from neuronal antigens when microglia cells phagocytose degenerated dopaminergic neurons or α-synuclein proteins that may be secreted by neurons. A specific example is observed from CD4+ T helper 17 (Th17) cells mediating neuronal death through the upregulation of the interleukin-17 (IL-17) receptor (IL-17R) and the secretion of IL-17. This leads to the upregulation of nuclear factor-κB (NFκB) within the neuron that mediates neuronal death. (**F**) CD4+ T lymphocytes can be activated by B cells that present neuronal peptides on the MHC II. CD4+ T cells can also activate B cells, causing them to produce antibodies or autoantibodies, which may induce neuronal death when they bind directly to neuronal antigens on dopaminergic neurons or when they bind to microglia receptors, activating and inducing the microglia to produce proinflammatory cytokines.

Our immune system interacts with the internal and external environment to maintain homeostasis. The seminal finding of activated microglia in PD brains established the link between inflammation and PD [22,23]. Thereafter, numerous preclinical and clinical studies confirmed enhanced inflammatory responses in the development and progression of PD. In addition, chronic inflammation has also been identified to be a key driver of progressive neurodegeneration. This arises from the activated phenotype of microglia that may be primed by ongoing systemic inflammation in PD patients [24]. Here, we will examine how innate immune cells induce neurodegeneration through immunoexcitotoxicity, the complement pathway and inflammasome activation (Figure 1). This will be followed by a discussion on possible immune-mediated neuronal death mechanisms involving lymphocytes of the adaptive immunity (Figure 1).

### 2.1. Immunoexcitotoxicity

Immunoexcitotoxicity, or inflammation-driven excitotoxicity, was postulated to have a role in PD neurodegeneration [25]. Excitotoxicity is a pathological event which induces cell death through the overstimulation of glutamate receptors such as the *N*-methyl-d-aspartate receptor (NMDAR) and alpha-amino-3-hydroxy-5-methyl-4-isoxazolepropionic acid receptor (AMPAR) [26,27]. Overactivation of these receptors, especially the calcium permeable AMPAR, are more excitotoxic as it excessively increases intracellular calcium levels [15,25,28]. There is considerable evidence that excitotoxic mechanisms participate in nigral death in PD-related pesticide, toxin and Parkin models [29,30]. Inflammatory processes from microglia activation are responsible for increasing excitatory glutamate receptor density and glutamate levels through the release of cytokines [25,31]. TNF-α and IL-1β were found to increase the production of excitotoxic glutamate by modulating glutaminase and inhibiting glutamate transport proteins, thereby elevating levels of extracellular glutamate [32,33]. These proinflammatory cytokines, together with other mediators such as inducible nitric oxide synthase, cyclooxygenase 2 (COX2) and IL-6, can also indirectly affect neuronal excitability [25]. TNFα and IL-1β both enhance neuronal sensitivity to glutamate and neuronal excitability by modulating the function and expression of inhibitory and excitatory receptors on synapses [31,34,35,36]. Consequently, the synergistic effect of IL-1β and TNF-α causes neurotoxicity through excessive nitric oxide production [37]. These neuroinflammatory processes may predispose the brain’s milieu to excitotoxicity. However, this begs the question whether excitotoxicity can in turn exacerbate neuroinflammation, leading to a vicious cycle that induces chronic inflammation. It is noteworthy that excitotoxicity often arise secondary to specific triggers such as α-synuclein, PD-related toxins and mitochondrial dysfunction [38,39,40]. With the presence of these perturbations in PD patients, we postulate that immunoexcitotoxicity might be one of the main mechanisms of neurodegeneration. Hence, the use of glutamate receptor antagonists to modulate excitotoxic pathways should be considered in light of other therapeutics targeting dysfunctional pathways in PD. It is also pivotal to adopt a multi-targeted approach to regulate both excitotoxic and inflammatory processes without affecting other pathways in the brain. This could be addressed through an in-depth study of specific pathways governing glutamate homeostasis and neuroinflammation in glial cells.

### 2.2. Pyroptosis

Pyroptosis is a form of programmed cell death induced by inflammatory caspases of the inflammasome pathway [41]. Similar to immunoexcitotoxicity, which is driven and exacerbated by a cytokine mediated process, pyroptosis creates a proinflammatory milieu with an increase in IL-1β and IL-18 levels. Inflammasomes are intracellular multiprotein complexes that mediate the maturation of IL-1β and IL-18 through the action of caspase 1. Inflammasomes are normally activated in the presence of damage-associated molecular patterns (DAMPs) or pathogen-associated molecular patterns (PAMPs) [42]. This triggers a cascade of inflammatory processes, contributing to neuronal injury and rapid cell death. Inflammasome activation in PD patients is evident from the high levels of IL-1β and the cytosolic nod like receptor protein 3 (NLRP3), correlating positively with α-synuclein levels [43,44]. Fibrillar α-synuclein is recognized as a potential DAMP, which activates the inflammasome through its interaction with the toll-like receptor 2 (TLR2) [45,46,47,48,49]. Gordon et al. (2019) reported that the long-term use of the NLRP3 inhibitor MCC950 protected against dopaminergic degeneration and abrogated α-synuclein pathology in mice [50]. Likewise, using a mouse model, Zhang’s team reported that the use of Salidroside, a bioactive compound from *Rhodiola resea* L., prevents NLRP3-dependent pyroptosis induced by 1-methyl-4-phenyl-1,2,3,6-tetrahydropyridine (MPTP) [51]. α-synuclein is also able to bind to toll like receptor 4 (TLR4) to activate microglia and astrocytes and it will be of interest to determine whether the interference of α-synuclein interaction with TLR4 has a role in inhibiting inflammasome activation [52]. While the drugs were effective in the animal model, it is still unclear whether pyroptosis actually induces nigral death in PD patients [53]. Therefore, studies will be required to validate and determine the mechanism underlying inflammasome mediated neurodegeneration [50]. Assessing the translational potential of the aforementioned immunomodulatory candidates should also be pursued through clinical trials with our urgent need for promising disease modifying therapeutics in PD.

### 2.3. Complements

The complement system is an ancient branch of innate immunity consisting of recognition molecules that have evolved to detect ‘foreign’ structures and recruit a cascade of protease enzymes and substrates to neutralize the pathogen. This is accomplished by the activation and recruitment of phagocytes, opsonization for phagocytosis and the formation of the membrane attack complex (MAC) [54]. The presence of early stage (C3d and C4d) and late stage (C7 and C9) complement proteins in the Lewy bodies of PD patients suggests the possibility of complement-mediated neuronal destruction [55]. Future work to determine whether neuronal degeneration and subsequent neuronal lysis of dopaminergic neurons may be a consequence of MAC formation is required [56]. Additionally, modulation of complement receptor 3 (CR3) in a PD pesticide mouse model through genetic deletion or the blockade using a CD11b antibody was found to ameliorate dopaminergic neurodegeneration. CR3 was reported to activate microglia NAPDH oxidase (Nox2) activation through the Src and extracellular regulated protein kinases (Erk) pathway [57]. This highlights the potential of targeting complement receptors in PD and compels further investigation on processes precipitating complement activation. The contribution of the complement system in PD is also evident from whole-exome sequencing analyses that identified mutations in the CUB and Sushi multiple domains 1 (*CSMD1*) gene in patients with familial late onset PD. CSMD1 encodes a complement control protein that activates complements and inflammation in the CNS [58]. This implies that dysregulation of the complement system influences the development of PD. More work on the interaction of complements with other PD-specific immune players may shed light on the pathways that can be targeted to abrogate complement-mediated neurodegeneration.

## 3. Mechanisms of Neuronal Death: Adaptive Immune System

The adaptive immune system, comprising B and T lymphocytes, forms the second arm of the immune system and has the ability to mount specific responses against foreign antigens. The presence of CD8+ and CD4+ T cells in the SN of PD patients strongly suggests a role of these subsets in neuronal death [19]. T lymphocytes transmigrate into the brain through the choroid plexus of the blood–CSF barrier and the post capillary blood brain barrier [59]. In the brain, major histocompatibility complex (MHC) presentation of PD-related (auto-) antigens, activation of cell death pathways and neuronal response to cytokines promote T cell-mediated neuronal death. MHC class I (MHC I) expression on catecholaminergic neurons in the SN and locus coeruleus allows neurons to be targets for CD8+ cytotoxic T cells (CTL) [60,61,62]. Moreover, upregulation of MHC class II (MHC II) on microglia in PD SN may also activate CD4+ T cells that can drive PD-specific antibody responses or inflict deleterious effects through the Fas ligand pathway [19,22,63]. Unfortunately, the nature of peptides presented on MHC I of dopaminergic neurons and MHC II of microglia cells is still unknown. Hence, further dissection of T cell activation due to peptide/antigen will reveal pathways that can be targeted to prevent neuronal damage. An elegant study performed by Sommer et al. (2018) highlighted the direct impact of PD derived T cells on dopaminergic neurons [64]. Using autologous co-cultures of T lymphocytes and induced pluripotent stem cell (iPSC)-derived midbrain neurons from PD patients, they revealed that neuronal death was attributed to IL-17 receptor upregulation and nuclear factor kappa B (NF-κB) activation. In addition, secukinamab, an FDA-approved anti-IL-17 antibody, successfully prevented neuronal death in these ex vivo cultures [64]. Their study is one of the first illustrating the mechanistic link between T cell mediated neurotoxicity and dopaminergic neurodegeneration. To bridge the gap between the physiological environment in the brain and their ex vivo system, inclusion of human iPSC derived microglia may clarify the role of glial cells. Alternatively, the use of three-dimensional (3D) brain organoids co-cultured with microglia-like cells presents an exciting option to improve disease modeling and evaluate the effects of immune cells on neurons [65,66]. Finally, discerning T cell’s antigenicity may explain the vulnerability of dopaminergic neurons in PD.

B lymphocytes, the key players in humoral immunity, are reliant on signals from CD4+ T helper cells for their antibody secreting function. At present, immunoglobulins, but not B lymphocytes, have been reported in the brains of PD patients [67]. This discovery sparked interest in the role of humoral immunity in PD pathogenesis. In 1998, the injection of purified immunoglobulin from PD patients into the SN of rats led to the loss of dopaminergic neurons, persistent perivascular inflammation and microglia infiltration [68]. Subsequently, studies sought to identify PD-specific autoantibodies as a diagnostic biomarker to prognosticate risk and disease progression. PD autoantibodies that recognize modified dopamine, neuronal proteins and α-synuclein have been identified, but the significance of these antibodies on dopaminergic neuronal death remains to be elucidated [69,70,71]. Without this knowledge, it will be difficult to harness the right approach to prevent autoantibody-mediated damage. Possible mechanisms of autoantibody-mediated neuronal death include its ability to neutralize neurotrophic factors, recruit complement factors, induce antibody dependent cellular cytotoxicity or internalize critical receptors into cells affecting neuronal function [72]. This emphasizes the need to determine the autoantibody targets and whether these autoantibodies in PD are directly or indirectly involved in the nigral neuronal death.

## 4. Potential Factors Triggering Immune Responses in PD Patients

### 4.1. Genetic Factors: Functional Role of PD Risk Genes in the Immune System

Over the past two decades, several genes have been identified as genetic risk factors in the development of PD. Familial PD accounts for approximately 10% of cases, while monogenic PD contributes to 30% of familial and 3–5% of sporadic cases [73]. Monogenic PD is associated with a single mutation in a dominantly or recessively inherited gene. Gene mutations in α-synuclein (*SNCA*), ubiquitin carboxyl-terminal esterase L1 (*UCHL1*), Grb-10 interacting GYF protein-2 (*GIGYF2*) and leucine-rich repeat kinase 2 (*LRRK2*) genes are inherited in an autosomal dominant manner, while mutations in PRKN (*Parkin*), phosphatase and tensin homolog (PTEN)-induced kinase 1 (*PINK1*), protein DJ-1 (*DJ-1*), ATPase 13A2 (*ATP13A2*) and A2 phospholipase group VI (*PLA2G6*) cause autosomal recessive PD [73,74]. Recent studies demonstrating the influence of certain PD-related genes on autoimmune development and immunomodulation further underscore the role of the immune system in the onset and progression of PD.

#### 4.1.1. LRRK2

LRRK2 mutations are the most common cause of dominantly inherited PD, with roles in regulating innate mediated inflammatory pathways. LRRK2 protein is expressed in immune cells and its expression is regulated by immune stimulation [75]. Induction of LRRK2 protein expression was observed in the presence of an inflammatory stimulus by lipopolysaccharide (LPS) or IFNγ [76,77]. Of note, LRRK2 inhibition was found to abrogate TNFα secretion and the induction of nitric oxide synthase in TLR4 stimulated microglia cells [76]. *LRRK2* knockdown in murine microglia similarly impaired LPS mediated inflammatory responses evidenced by reduced p38 mitogen activated protein kinase phosphorylation and NFκB activity [78]. PD patients with G2019S and R1441G *LRRK2* mutation had elevated levels of basal cyclooxygenase-2 RNA levels in fibroblasts, which were significantly reduced with *LRRK2* silencing [79]. The association of LRRK2 to inflammation is also supported from reports linking *LRRK2* polymorphisms to inflammatory conditions such as leprosy and inflammatory bowel disease (IBD) [80]. Collectively, these studies demonstrate LRRK2’s role in regulating inflammation and underscore the potential of LRRK2 inhibitors as a therapeutic target. Interestingly, Shutinoski’s team revealed that the control of LRRK2 kinase activity in immune cells was vital in countering specific pathogens [81]. They reported that G2019S *LRRK2* mice with increased kinase activity conferred greater protection against *Salmonella typhimurium* through myeloid-induced production of reactive oxygen species and chemotaxis augmentation. Surprisingly, the infection of G2019S *LRRK2* mice with reovirus causing invasion of the nervous system led to greater mortality from encephalitis in females. On the other hand, the kinase inactive control, D1994S *LRRK2* mutant mice had a higher survival rate due to reduced LRRK2 autophosphorylation with reovirus infection [81]. Although this study was performed in the context of a specific infection, it highlights the potential of modulating LRRK2 kinase activity as a therapeutic in PD patients with monogenic *LRRK2* mutation. Additionally, Shutinoski’s work adds to mounting evidence that LRRK2 has critical roles in immune cells. However, the immune cell type responsible for promoting LRRK2 related PD has yet to be identified. Knowing the precise immune targets may reduce the risk of unintended effects of LRRK2 inhibitors on other immune cells and systems where LRRK2’s function is crucial. Additionally, epidemiological studies on whether previous infections play a role in the development of PD in *LRRK2* carriers compared to healthy *LRRK2* carriers, will substantiate the ‘multiple hit’ hypothesis in PD [80,82]. Analyzing downstream targets of LRRK2 variants that regulate inflammatory responses will aid in our search of useful candidates for immune modulation. Considering that LRRK2 is implicated in chronic diseases such as PD, leprosy and IBD, the role of LRRK2 in chronic inflammatory models and autoimmunity warrants further investigation. Tackling these unanswered questions serves to inform us of potential interventions in LRRK2-related conditions.

#### 4.1.2. *HLA* Genes

Genome-wide association studies and fine mapping of genes are useful in the identification of the risk or protective genetic variants in PD. The MHC on chromosome 6, which houses the *HLA* genes, contributes to an individual’s susceptibility to PD. The *HLA* gene is highly polymorphic with variations in different ethnic and geographic populations. HLA class I gene products present intracellular antigens to cytotoxic CD8+ T cells, while HLA class II gene products present extracellular peptide fragments to CD4+ T helper cells [83,84]. HLA-DRB1*0301 was noted to be overrepresented in British PD patients and is a risk allele in the Chinese Han population [85,86]. Association of other *HLA I* and *II* alleles including HLA-A*2, HLA-B*17 and B*18, HLA-DQB1*06 have also been reported [87]. Different *HLA* alleles are responsible for the variation in immune responses among individuals [88]. A deep sequencing study of *HLA* genes carried out to associate single nucleotide polymorphisms (SNP) with the risk of PD revealed genetic variant protection by valine at position 11 and a specific epitope at position 70–74 on the HLA-DRB1 molecule of HLA-DRB1*04:01, *04:04 and *04:05. Conversely, the absence of valine at position 11 in individuals with the specific epitope present in HLA-DRB1*01:01 and *01:02 contributes to the risk of PD development and this is modified with a history of smoking. An α-synuclein peptide binding prediction to *HLA-DRB1* alleles was performed and greater binding affinity was predicted in the presence of the risk alleles containing the specific epitope without valine at position 11 [84]. More of such studies will guide our understanding of autoimmune mechanisms in PD and prompt further exploration to modulate specific peptide affinity in individuals with PD genetic risk variants.

### 4.2. Environmental Factors: Double Hit Theory

#### 4.2.1. Gut-Brain Axis

The presence of Lewy body inclusions in extra-nigral sites such as the enteric plexus of the stomach and the olfactory bulbs (OB) accounts for some non-motor symptoms during the prodromal period in PD patients. The double hit theory proposes that a neurotrophic pathogen enters the brain through the nose and the gut, travels anterogradely or retrogradely through neurons to reach the SN and cause PD [89]. Notably, the vagal nerve has also been identified to enable the transfer of the pathological form of α-synuclein from the gastrointestinal tract to the brain [90]. Using a novel gut-to-brain α-synuclein transmission model, truncal vagotomy and α-synuclein deficiency effectively attenuated PD-associated neurodegeneration in these mouse models [90]. These findings might explain the data from cohort studies, reporting a lower risk of PD development in individuals who had truncal vagotomy [91]. Research on the gut-brain axis in neurodegeneration was fueled in part by the observation that approximately 80% of PD patients experience constipation [92]. Emerging data inform us of the bidirectional communication of the gut and brain, as well as the presence of microbiota dysbiosis in PD patients affecting intestinal inflammation and α-synuclein aggregation [93]. However, the nexus between intestinal changes in PD and neurodegeneration still lacks experimental support. Clues on the role of colonic inflammation in PD pathogenesis have been suggested from association studies reporting a 22% increased risk of PD in patients with IBD [94]. Interestingly, early exposure to anti-TNF therapy in IBD patients substantially reduced PD incidence by 78%, reinforcing the influence of systemic and gut-specific inflammation in PD development [95]. T cells may be implicated as a mediator between intestinal immunity and dopaminergic neuronal death. Campos-Acuña et al. (2019) postulated that the gut may trigger autoreactive T cells by providing structures similar to autoantigens via molecular mimicry or activate T cells through the production of metabolites [96]. Factors that enable a greater accumulation of enteric phosphorylated α-synuclein (p-αsyn) in the presence of a proinflammatory gut milieu and gut dysbiosis may explain how inflammation drives protein aggregation. The expression of the M3 muscarinic receptor (M3R), which may have a role in modulating the permeability of the epithelial barrier, was reduced in PD patients. M3R expression was negatively correlated to the number of small sized p-αsyn aggregates [97]. Although the significance and cause of the change are not well understood in PD patients, it might serve as a lead in deconstructing processes, contributing to α-synuclein aggregation in the gut. Another hallmark study by Sampson’s team highlighted the involvement of the gut microbiota in neuroinflammation and the induction of PD motor symptoms [98]. α-synuclein overexpressing mice harboring complex microbiota had increased α-synuclein aggregation and microglia activation. Fecal transplantation from PD patients into these mice enhanced motor deficits compared to transplants from healthy donors, providing support that both gut dysbiosis and genetic susceptibility influence disease outcomes [98]. Taken together, a myriad of factors, such as microbiota, metabolites and receptors, are critical for the maintenance of intestinal immunity. Hence, studies to identify protective microbes or metabolites capable of reducing the inflammatory milieu in PD patients or reversing their gastrointestinal disturbance may be beneficial in mitigating PD development.

#### 4.2.2. Olfactory Involvement

Olfactory impairment is an early sign of PD, which affects 82–90% of PD patients. The majority of patients experience hyposmia with deficits in odor identification, detection, memory and discrimination [99]. The olfactory network is complex and communicates directly to the SN through multisynaptic transmission [100]. Evidence of the OB as a site of α-synuclein propagation was seen from Rey’s study demonstrating the transneuronal spread of α-synuclein aggregates after the injection of α-synuclein fibrils into the OB of mice [101]. Recent work identifying the abundance of α-synuclein inclusions in non-neuronal cells of the anterior olfactory nucleus, such as the microglia, pericytes and astrocytes in PD patients, supports the possible involvement of the OB in PD progression [102]. Although the mechanism of olfactory dysfunction remains poorly understood, recent work by Nui’s team suggested that OB inflammation induces α-synuclein pathology, enabling its spread and the development of PD [103]. They found that intranasally administered LPS-induced inflammation by activating microglia. Activated microglia enhances IL-1β secretion and increases levels of phosphorylated and total α-synuclein in the OB. A concomitant loss of dopaminergic neurons in the SN and motor impairment of LPS treated mice was observed. These PD-related changes were abrogated in the presence of minocycline used to inhibit microglia activation and in mice with IL-1 receptor type I (IL-1R1) deficiency [103]. This study provides the first evidence that IL-1R1 is necessary for LPS induced α-synuclein pathology.

Knowing that the gut and OB are exposed to various insults from the external environment, the challenge lies in understanding whether these sites are an initial point of pathology or whether changes observed are secondary to ongoing pathogenic processes in PD. Identifying predisposing, precipitating and propagating factors of inflammation in the olfactory and gut microenvironment allows specific signaling pathways to be studied in the context of environmental toxin or pathogens. This will guide our approach in preventing PD progression by treating the sites which are affected by PD pathology at an early stage of the disease.

### 4.3. Peptide-Specific T Cells: Potential Biomarker, Grievous Effect or Both

#### 4.3.1. α-Synuclein-Specific T Cells

The seminal discovery of α-synuclein-specific T cells in PD patients contributes to our understanding of autoimmune mechanisms in PD pathogenesis. Sulzer et al. (2017) identified T cells recognizing antigenic regions of α-synuclein [62]. T cell responses were primarily mediated by IL-5- or IFNγ-producing CD4+ T cells and IFNγ-producing CD8+ T cells. Specific α-synuclein epitopes from the Y39 region exhibited good binding affinity to specific HLA class II variants DRB1*1501 and DRB5*01:01 [62]. More significantly, α-synuclein-specific T cells were detected in a PD patient more than a decade before his PD diagnosis [104]. This finding is consistent with the proinflammatory state observed in PD patients and raises further question on whether these cells have a grievous effect on dopaminergic neuronal death before the onset of the motor symptoms. Longitudinal studies of healthy individuals will shed light on the utility of α-synuclein-specific T cells as a guiding biomarker to catch individuals at risk or in the early stages of PD. Given that the α-synuclein pathology is also implicated in other α-synucleinopathies, such as Lewy body dementia or multiple system atrophy, comparing α-synuclein-specific T cell reactivity will unravel the usefulness of the marker as a surrogate for neuronal loss and neuroinflammation. Although this suggests that the marker may not be PD-specific, preclinical diagnosis of PD should take other clinical, biochemical and imaging markers into consideration, similar to the diagnostic criteria used for the diagnosis of an autoimmune condition. In Lindestam’s recent study, a higher magnitude of α-synuclein reactivity was observed in PD compared to healthy controls [104]. However, a significant difference was only noted in IL-10 T cell responses, but not with IL-5 and IFNγ T cell responses in PD patients. A pool of 11 α-synuclein epitopes was used in this study and not restricted to a specific haplotype [104]. Although the epitopes were not clearly defined, this highlights the value of studies creating α-synuclein peptide pools, consisting of a wide range of predicted peptides that are associated with different *HLA* allele variants. This increases the chance of detecting α-synuclein-specific T cells in at-risk individuals with a specific *HLA* allele variant. Notably, a positive correlation of α-synuclein T cell reactivity was found in those with a low Levodopa equivalent dose (LED), age (older than 70 years) and time since diagnosis (<10 years) in PD patients. The combination of these three variables gave a 68% specificity and 54% sensitivity in this cohort of patients. The consideration of other PD-specific antigens and proper categorization of patients according to the duration of their disease may improve the sensitivity of using T cells as a biomarker [104]. These cells can either cause grievous harm to dopaminergic neurons or serve as a marker indicating the ongoing neuronal death secondary to α-synuclein accumulation. Discriminating the function of α-synuclein-specific T cells creates new venues to interfere with T cell responses, thereby mitigating disease progression.

#### 4.3.2. Mitochondrial Peptide-Specific T Cells

Mitochondrial dysfunction is recognized to be a central factor in PD pathophysiology, affecting both sporadic and familial PD patients. In familial PD, several PD-related genes such as *SNCA*, *LRRK2*, *Parkin*, *PINK1*, *ATP13A2*, vacuolar protein sorting 35 ortholog (*VPS35*) and coiled-coil-helix-coiled-coil-helix domain containing protein 2 (*CHCHD2*) have been identified to directly affect the mitochondria [105]. PINK1 and Parkin both govern mitochondrial quality control and have been implicated in the regulation of adaptive immunity [106]. The absence of the PINK1 and Parkin was found to induce mitochondrial antigen presentation on MHC I molecules in antigen presenting cells through the formation of mitochondria-derived vesicles [107]. This groundbreaking discovery of PINK1 and Parkin’s role in the repression of mitochondrial antigen presentation supports a non-cell autonomous model where cytotoxic T cells contribute to dopaminergic neuronal destruction. This was further validated in a recent study, where an intestinal infection in *PINK1* knockout mice (*PINK1-/-*) induced mitochondrial antigen presentation and the establishment of mitochondrial-specific CD8+ T cells [108]. Subsequent infiltration of these cytotoxic T cells in the brain leading to nigral death explained the emergence of motor impairment in *PINK1-/-* mice. The concomitant recovery of the PD behavioral phenotype and density of the tyrosine hydroxylase signal was associated with a reduction in the levels of mitochondrial-specific T cells [108]. These findings provide new lines of evidence that genetic susceptibility may contribute to autoimmune mechanisms involved in PD pathogenesis. With the insights afforded by the current study, it is worth investigating the contribution of PD-related genes to the development of PD antigen-specific T cells, especially in individuals with genetic perturbations. Although the identification of disease-specific T cells is key for early disease diagnosis, the extent of antigen-specific T cells in neuronal destruction needs to be interrogated. As we strive to integrate our understanding of genes and its role on immune cells, there is a need to adopt a system-based approach when genes are studied in the context of a specific environmental trigger.

## 5. Gaps and Future Studies

A substantial body of evidence suggests that the immune system is involved in PD pathogenesis. However, the search for an appropriate biomarker, a specific immune target implicated in the initiation and propagation of PD and the effect of gut dysbiosis on immune cells in PD requires further investigation.

Currently, diagnostic tests for early detection of PD that are accurate and cost effective are lacking [109]. Serum biomarkers through immunophenotyping, cytokine profile analysis and the presence of autoantibodies have been extensively investigated, though an authentic biomarker has yet to be identified. Current studies are confounded by differences in patient populations, disease stage, patient comorbidities, medication influence, sample processing and storage and methodology for analyses [14]. Aside from the discovery of α-synuclein autoantibodies, which may have pivotal roles in disrupting α-synuclein aggregation, the search for other PD-specific autoantibodies have been largely inconclusive. Reports on autoantibodies have been observational and autoantibody validation has been inconsistent [110]. A large-scale population-based screening of autoantibodies using different protein array platforms on the same samples may unravel novel autoantibodies. The use of independent cohorts or subsets of patients who may have similar symptomology will aid in the correlation of clinical parameters with the presence of specific autoantibodies. Deciphering autoantibody isotypes enables specific downstream effector functions to be investigated. Importantly, elucidating the functional effect of the autoantibody on its protein target will guide the development of potential therapeutics to counter or promote the antibody effect.

Despite extensive work on immune cells contributing to neuroinflammatory processes in PD, a specific immune target responsible for neuronal degeneration has yet to be reported. The involvement of innate and adaptive immune cells and its variable responses to different PD-related triggers add to the complexity. Critical to the identification of the immune cell involved in PD pathogenesis, immunophenotyping should be performed on healthy individuals at high risk of PD development due to genetic factors, the presence of prodromal non-motor symptoms and those exposed to PD-associated environmental toxins. With our knowledge that immune subsets may change at different stages of the disease, phenotyping patients at an early or late stage or when they are treatment naïve and post treatment enables the treatment or disease-related changes to be accounted for. Ascertaining the primary immune player for early intervention enables the development of an effective immunotherapy to thwart the neurodegenerative process.

The gut is greatly influenced by diet, the environment, drugs and genetics [111]. Although mediators produced by the gut microbiota have been implicated as a possible trigger of immune responses to antigens derived from Lewy bodies, it is still unknown whether these changes are a cause or an effect of PD [96]. Hence, a systematic approach by carefully defining an individual’s genetic risk factors and extrinsic exposure in different cohorts will aid in the identification of microbes or metabolites involved in PD pathology in specific groups of PD patients. Comparing gut dysbiosis in PD and healthy individuals with a high risk of PD development will also shed light on gut-related changes that may be causal or consequential to PD development. Finally, mechanistic studies on factors compromising the gut-brain axis by inflammation will guide our approach to treat and screen healthy individuals at risk of PD.

**Figure 2:**
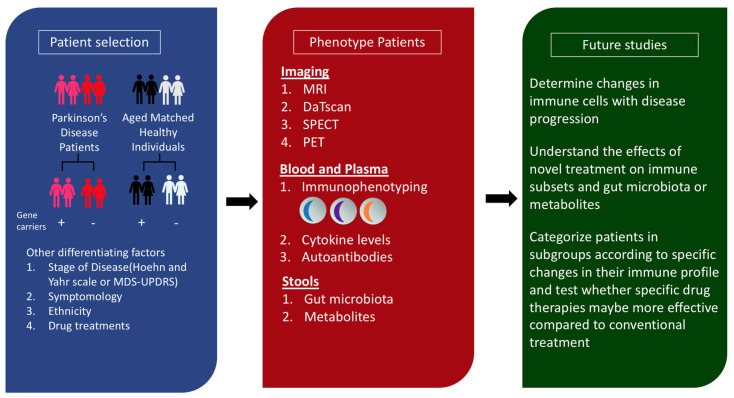
Proposed framework to integrate the study of genes, gut dysbiosis and immune cells in patient cohorts. Precise patient selection by identifying patients who are carriers of risk alleles or familial PD related genes allows the effect of genes on the immune system to be investigated. Other patient variables, such as (1) stage of the disease determined by Hoehn and Yahr scale or the movement disorder society-unified Parkinson’s disease rating scale (MDS-UPDRS), (2) motor or prodromal non-motor symptoms, (3) ethnicity and (4) drug treatments, can be considered to characterize patients into specific subgroups. Next, several modalities can be used to determine the baseline immune profile of patients and healthy individuals. Imaging using magnetic resonance imaging (MRI), dopamine transporter scan (DaTscan), single photon emission computed tomography (SPECT) and positron emission tomography (PET) can also be performed. Blood and plasma-based assays can be conducted to understand changes in immune subsets, cytokine levels and the presence of disease-specific autoantibodies. The collection of stools enables the analysis of stool microbiota and metabolites to be performed. Future studies can facilitate the monitoring of immune changes with disease progression, in the presence or absence of novel therapeutics, and provide information to classify patients in groups for personalized medicine.

In essence, we need a holistic multi-disciplinary integrative approach to study the immune profiles and gut perturbations in PD patients (Figure 2).

## 6. Conclusions

**Figure 3:**
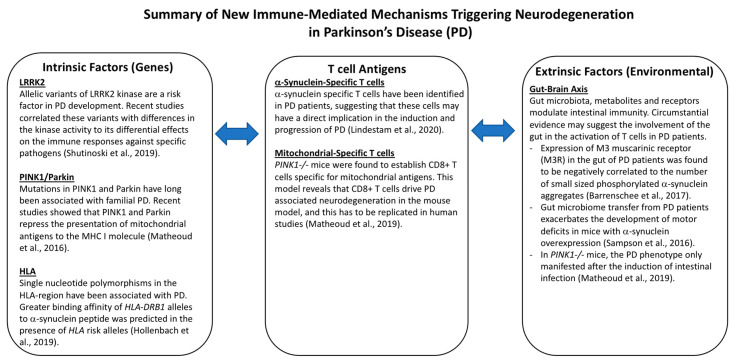
Summary of New Immune-Mediated Mechanisms Triggering Neurodegeneration in Parkinson’s Disease.

There are still many challenges in understanding the multifaceted neuroinflammatory mechanisms inducing dopaminergic neuronal death in PD. While there are suggestions that various immune components are implicated in PD pathogenesis, the cause and effect association remains unclear. This interplay is further complicated by unknown relationships between extrinsic triggers (e.g., gut dysbiosis) and intrinsic changes in immune cells (e.g., genetic mutations). As we elucidate the complex interactions, understanding the mechanisms of neuronal death will be key in our quest to develop targeted cell therapies to halt disease progression. Current observations suggest that there appears to be an interplay of immune mechanisms, genes, gut immunity and antigen-specific T cells, contributing to dopaminergic neurodegeneration (Figure 3). Proper validation and mechanistic studies may help in the identification of PD-specific autoantibodies that can serve as an early predictor of the disease. Finally, adopting a comprehensive immune profiling and gut dysbiosis characterization in healthy individuals and PD patients categorized according to their risk factors is a promising approach for identifying therapeutic targets. Altogether, early identification of PD and personalized immune modulation according to the patient’s immune signature is likely the way forward against this debilitating condition.
